# Innate immune defects in HIV permissive cell lines

**DOI:** 10.1186/s12977-016-0275-8

**Published:** 2016-06-27

**Authors:** Antonio Rausell, Miguel Muñoz, Raquel Martinez, Thierry Roger, Amalio Telenti, Angela Ciuffi

**Affiliations:** Clinical Bioinformatics lab, Imagine Institute, Paris Descartes University - Sorbonne Paris Cité, 75015 Paris, France; Institute of Microbiology, University Hospital of Lausanne (CHUV) and University of Lausanne, 1011 Lausanne, Switzerland; Infectious Diseases Service, Department of Medicine, University Hospital of Lausanne (CHUV) and University of Lausanne, 1011 Lausanne, Switzerland; Genetic Medicine, J. Craig Venter Institute, La Jolla, CA 92037 USA

**Keywords:** Innate immunity, HIV, Cell lines, TCR activation, RNA-Seq

## Abstract

**Background:**

Primary CD4+ T cells and cell lines differ in their permissiveness to HIV infection. Impaired innate immunity may contribute to this different phenotype.

**Findings:**

We used transcriptome profiling of 1503 innate immunity genes in primary CD4+ T cells and permissive cell lines. Two clusters of differentially expressed genes were identified: a set of 249 genes that were highly expressed in primary cells and minimally expressed in cell lines and a set of 110 genes with the opposite pattern. Specific to HIV, HEK293T, Jurkat, SupT1 and CEM cell lines displayed unique patterns of downregulation of genes involved in viral sensing and restriction. Activation of primary CD4+ T cells resulted in reversal of the pattern of expression of those sets of innate immunity genes. Functional analysis of prototypical innate immunity pathways of permissive cell lines confirmed impaired responses identified in transcriptome analyses.

**Conclusion:**

Integrity of innate immunity genes and pathways needs to be considered in designing gain/loss functional genomic screens of viral infection.

**Electronic supplementary material:**

The online version of this article (doi:10.1186/s12977-016-0275-8) contains supplementary material, which is available to authorized users.

## Background

 The success of viral replication depends on the ability of a virus to exploit host cell components and evade host cell defense and is thus affected by the specific cellular environment [1–3]. In the past decades, studies on HIV have often used established T cell lines to investigate the molecular mechanisms of viral replication and virus-host interactions because of ease of infection. In contrast, resting primary CD4+ T cells are difficult to infect and need to be activated through T-Cell Receptor (TCR) stimulation in order to enhance their susceptibility to HIV infection [4, 5]. Despite stimulation, infection efficiency of primary cells rarely reaches the level observed in T cell lines, suggesting that the cellular environment is different. Many transformed cell lines show global or narrow defects in the Interferon (IFN) response, including deficits in signaling (eg., JAK1, [6, 7]) and in expression of transcription factors (eg., ISGF3 and STAT1, [8–12]). A reduced responsiveness to IFN due to defects in the type I IFN pathway is also a common hallmark among malignant cells [13]. There is ample evidence of innate immunity defects in cell lines used in HIV research. For example, HEK293T do not express detectable levels of STING [14] or TLRs [15]. A link between antiviral defense and cell proliferation has been proposed for HEK293T and HeLa cells. Here, immune defects in the cGas-STING pathway may be explained by the constitutive expression of viral oncoproteins that interfere with the innate immune response while at the same time supporting cell transformation [16]. Although still responsive to IFN-α in terms of general ISG expression, Jurkat cells are unable to induce the expression of specific antiviral genes [17]. SupT1 and other cell lines exhibit a diversity of patterns in response to interferon [18]. Limited response to IFN-α and high permissiveness to HIV infection led to the identification of MX2 in CEM cells [19]. Thus, lack of integrity of the IFN pathway or the failure to induce antiviral factors is thought to underlie the ease of propagation of numerous viruses in cell lines [17].

We aimed at characterizing the transcriptional landscape of innate immunity genes in cell lines used in HIV research, to contrast them with primary CD4+ T cells, and to validate some of the defects in functional assays. The emerging picture is that of a diversity of patterns of downregulation that support the concept of innate immunity defects underlying the high permissiveness of commonly used cell lines to HIV.

## Findings

### Innate immunity gene expression in primary cells and in HIV-permissive cell lines

**Fig. 1:**
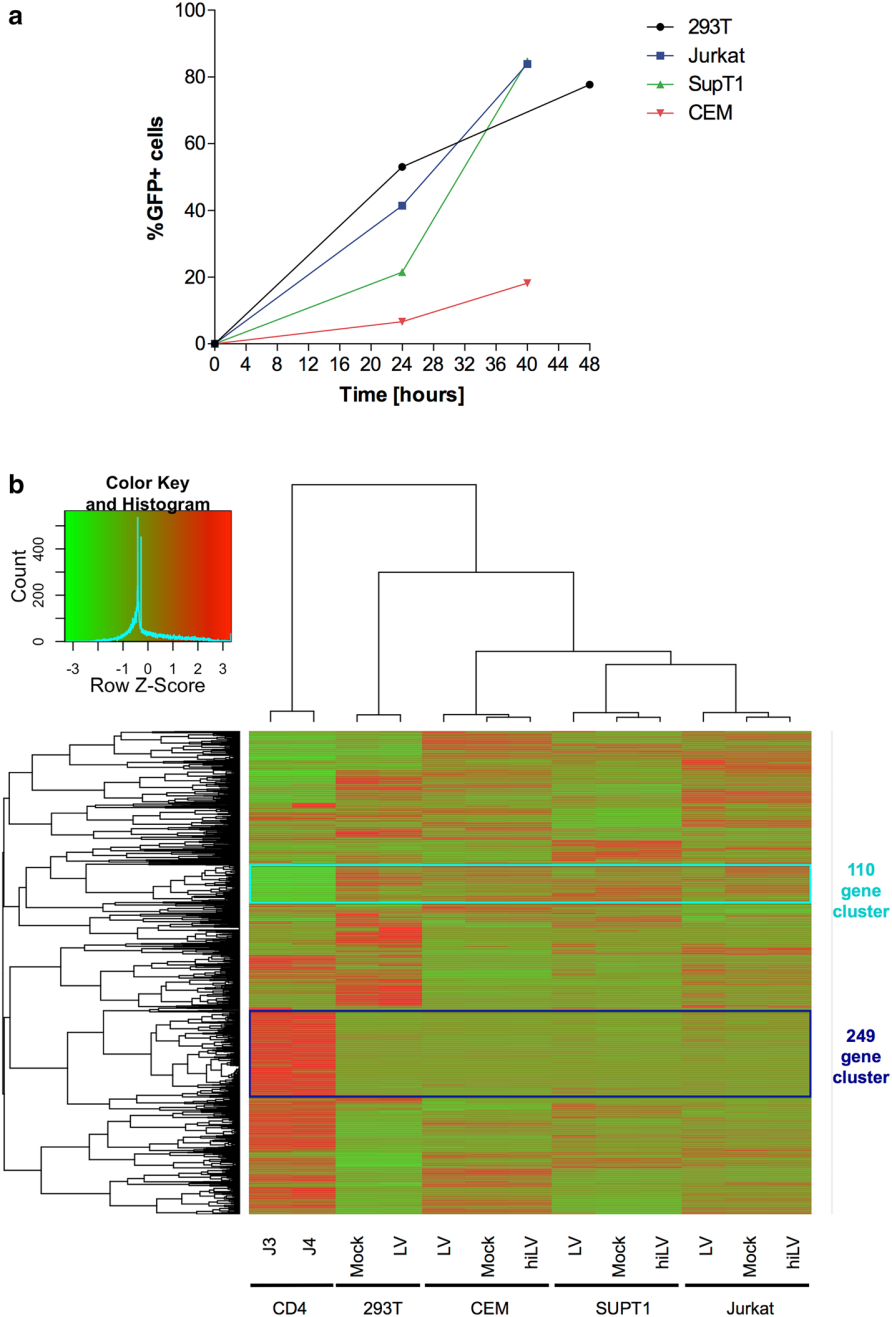
Differences between resting primary CD4+ T cells and laboratory cell lines in innate immunity. **a** Laboratory cell lines revealed differential permissiveness to HIV infection. Cells were infected using a VSV-G pseudotyped HIV virus. Viral infection success was assessed by FACS analysis of the expression of the virally encoded GFP reporter gene, and ranged from ~7 to 53 % according to the cell line infected. **b** Heatmap of expression values of innate immunity genes in resting CD4+ T cells and laboratory cell lines. The *figure* shows the expression values of 1473 innate immunity genes in resting CD4+ T cells from two donors (CD4_J3 and CD4_J4), and four human laboratory cell lines HEK293T, Jurkat, SupT1 and CEM. Cell lines were evaluated in 3 conditions: uninfected mock (Mock), heat-inactivated HIV vector (hiLV) and HIV vector-infected (LV). Complete hierarchical clustering of genes and cell samples was based on Pearson correlation of variance-stabilized read counts (Methods). *Color scale* indicated in the legend corresponds to z-scores of RPKM distributions per gene, ranging from *green* (low) to *red* (high) expression. Two prominent clusters of genes are highlighted: 249 genes with a high expression in resting CD4+ T cells and a low relative expression in all laboratory cell lines (*dark blue square*) and 110 genes with a low relative expression in resting CD4+ T cells and a high relative expression in all laboratory cell lines (*cyan square*). The genes within each of these two clusters are listed in Additional file 3: Table S2

To investigate the impact of innate immunity on HIV permissiveness, we analyzed the transcriptional state of 1503 innate immunity genes (previously defined in [20]; Additional file 1: Table S1) by whole transcriptome profiling of resting primary CD4+ T lymphocytes isolated from two different healthy blood donors on one hand, and the panel of four human cell lines on the other hand: 3 lymphoblastic T cell lines—SupT1, Jurkat and CEM- and the human embryonic kidney (HEK) 293T cell line (Methods). For cell lines, transcriptome of uninfected cells as well as cells infected with a competent or heat-inactivated VSV-G pseudotyped HIV vector were investigated. Of these, expression of 1473 innate immunity genes was detected in at least one sample and further analyzed. Cluster analysis of relative expression values grouped cell types according to their co-expression profiles in innate immunity genes (Fig. 1), recapitulating the principal component analysis on the whole transcriptome and in line with previous studies [21] showing that cell identity was the major determinant compared to the influence of HIV infection (Additional file 2: Figure S1).

To identify innate immunity genes associated with HIV permissiveness at the cellular level, we focused on genes that were differentially expressed in resting CD4+ T cells compared to cell lines. Two clusters corresponding to such criteria were identified. First, a cluster of 249 innate immunity genes that were highly expressed in primary cells and lowly expressed or absent in cell lines (Fig. 1, dark blue cluster, 204 of them with a fold change higher than 2, Benjamini–Hochberg adjusted p value <0.01, Methods). This set included Toll-like receptors (TLRs), NOD-like receptors (NLRs), sialic acid binding Ig-like lectin (SIGLEC) and C-type lectin (CLEC) family members, interleukins and their receptors (ILs and ILRs), chemokine ligands and receptors (CCLs, CXCLs and CCRs), caspases, complement components, MAP-kinases, transcription factors and regulators (Interferon -IFN- regulatory factors -IRFs, JAK/STATs, FOS and JUN) among others (Additional file 3: Table S2). The second cluster included 110 innate immunity genes (Fig. 1, cyan cluster, 101 genes with a fold change higher than 2, Benjamini–Hochberg adjusted p value <0.01) with low expression in resting CD4+ T cells and high expression in cell lines. This cluster contained genes acting as suppressors of the innate immune response (e.g. TYRO3, BIRC5), inhibitors of transcription factors (NKIRAS2 -inhibitor of NFKB- and PIAS4 -inhibitor of activated STAT4) and transcriptional repressors (HES4, CSDA, RCOR1) (Additional file 3: Table S2). Of note, excluding samples infected with HIV or exposed to heat-inactivated HIV from the analysis led to a highly similar clustering of genes (Spearman correlation of gene distances between the two clusterings of 0.96, p value <2.2e−16, Additional file 4: Figure S2), reproducing the same two clusters.

### Effect of CD4+ T cell activation on relevant innate immunity genes

**Fig. 2:**
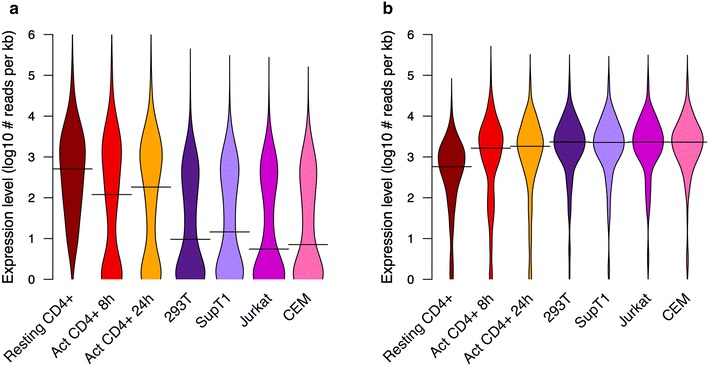
Activation of primary CD4+ T cells produces an intermediate expression phenotype in clusters of innate immunity genes differentiating resting CD4+ T cells from permissive cell lines. Distribution of expression levels of the 249 (**a**) and 110 (**b**) gene clusters represented as *violin plots*. Seven distributions are shown summarizing the average expression values in resting CD4+ T cells (*dark red*), activated CD4+ T cells at 8 h (*red*) and 24 h (*orange*) after TCR activation, and four human laboratory cell lines HEK293T (*pink*), Jurkat (*light violet*), SupT1 (*magenta*) and CEM (*dark violet*). *Lines* within the *plots* represent the median of such distributions. All distributions are significantly different from the resting CD4+ T cells in both *panels* (Wilcoxon rank sum test, Bonferroni p.adjusted <1E−3). Expression values on the y-axis represent the log10 transformation of the number of library size-normalized reads per kilobase of exonic sequence averaged within cell type (Methods)

We then inspected the impact of cell activation on the 249- and 110-gene clusters. Activated CD4+ T cells displayed an intermediate phenotype (Additional files 5, 6: Figures S3, S4). For the 249-gene cluster, median expression levels at 8 and 24 h (m = 2.08 and 2.26 log10 reads per kilobase, respectively) of TCR activation were between those of primary resting CD4+ T cells (m = 2.71 log10 reads per kilobase) and cell lines (m = 0.74–1.16 log10 reads per kilobase; Fig. 2a). For the 110-gene cluster, median expression levels at 8 and 24 h (m = 3.21 and 3.26 log10 reads per kilobase, respectively) of TCR activation were closer to those of cell lines (m = 3.36–3.37 log10 reads per kilobase; Fig. 2b) than to primary resting CD4+ T cells (m = 2.76 log10 reads per kilobase) (Fig. 2b). The distributions of the expression levels of activated CD4+ T cells and cell lines were in all cases significantly different from those of the resting CD4+ T cells (Wilcoxon rank sum test, Bonferroni p-adjusted <1E−3).

### Transcriptional and functional defects in innate immunity pathways in cell lines

**Fig. 3:**
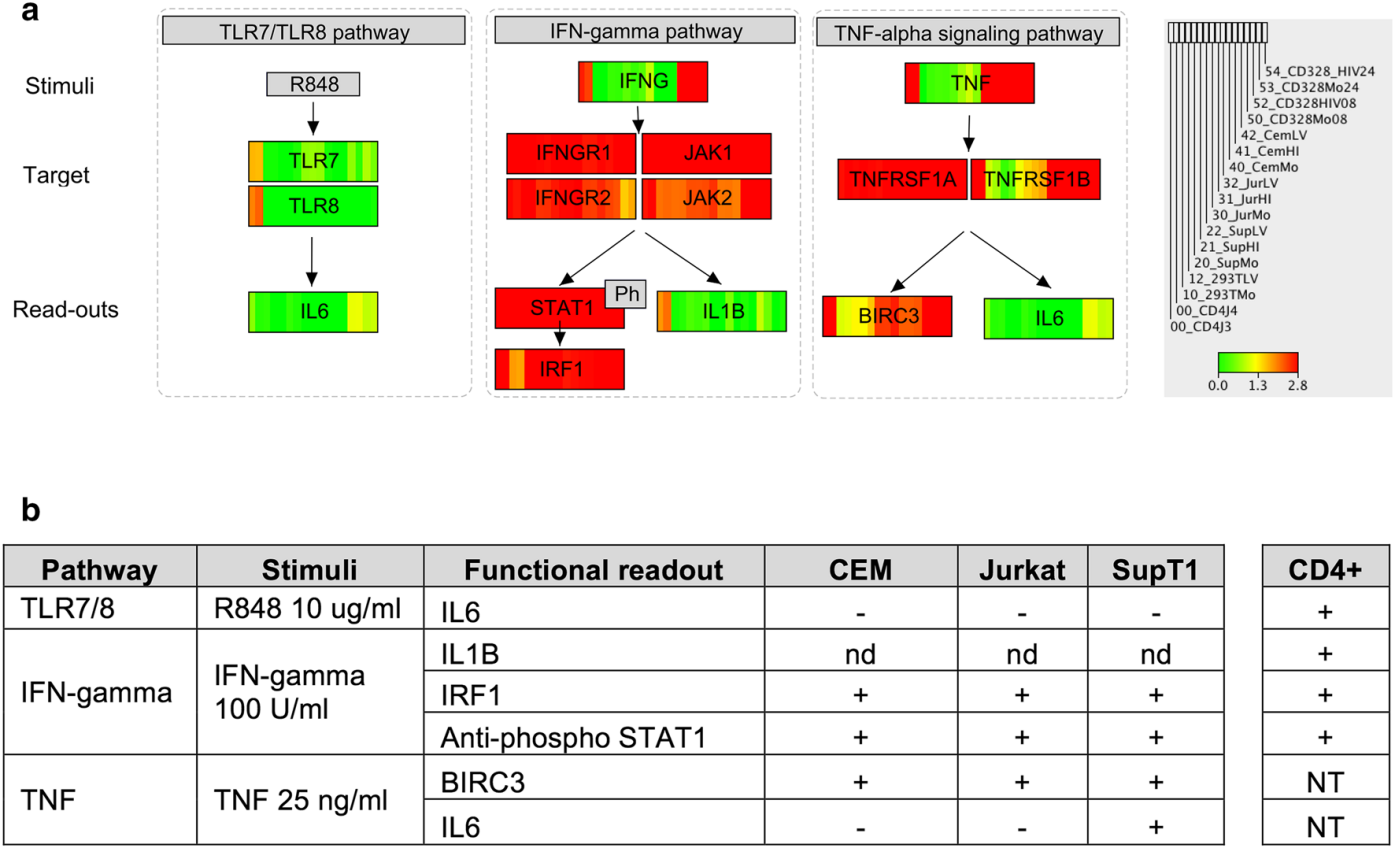
Defects in 3 selected innate immunity pathways in cell lines. **a** The *figure* represents a simplified view of the TLR7/TLR8, IFN-gamma and TNF-alpha signaling pathways. *Boxes* representing genes display the transcriptional levels detected in RNA-seq libraries of resting CD4+ T cells, the four human laboratory cell lines HEK293T, Jurkat, SupT1 and CEM -mock (MO), heat-inactivated (HI) and HIV-infected (HIV)- and 4 samples corresponding to Activated CD4+ T cells at 8 and 24 h after TCR activation. *Inset* describes the order of the libraries as well as the *color-code scale* of expression levels (log10 transformation of the number of library size-normalized reads per kilobase of exonic sequence) ranging from 0 (*green*) to ≥2.8 (*red*; lower limit of the 9th-decile of expression values). The expression levels indicated for IFNG and TNF convey the basal expression level before adding the stimuli. **b** Experimental validation of the functional integrity of the selected innate immunity pathways. The table reports the stimuli applied and the functional read-out measured 24 h after stimulation. A *positive sign* indicates positive detection of functional read-outs (transcript levels by RT-qPCR or phosphorylation of STAT1 by Western blot analysis). *NT* not tested, *nd* not detected

Transcriptional profiling pointed to expression defects in innate immunity genes suggesting impaired intracellular defense in cell lines. To tackle this possibility, we characterized transcriptional patterns along the signaling cascade (receptors, signal transduction, transcription factors or effectors). Analysis of the toll-like receptor (TLR) pathways showed that most receptors -including TLR7, TLR8 and TLR9- are minimally expressed in permissive cell lines and in activated CD4+ T cells (Additional file 7: Figure S5). However, downstream of the receptors, the signal transduction cascades appeared intact in terms of expression levels of their constituent genes. Differences between resting CD4+ T cells and cell lines were again identified at the level of expression of transcription factors (FOS and IRF5) and effectors (inflammatory cytokines and co-stimulatory molecules), with activated CD4+ T cells displaying intermediate phenotypes consistent with the results presented in Figs. 2, 3a and Additional file 5: Figure S3. Similar patterns were found in the IFN-gamma-signaling pathway (Additional file 8: Figure S6) and the TNF-alpha signaling pathway (Additional file 9: Figure S7). Here, genes involved in the signaling cascade appeared well expressed across cell types. However, transcriptional differences are observed for genes triggering the signaling (IFN-gamma, TNF-alpha and TNFRSF18) and effector genes (e.g. IFN-stimulated genes in the case of IFN-gamma pathway and IL6 or BIRC3 in the TNF-alpha pathway).

We used functional assays to evaluate the consequences of diminished expression of genes involved in those selected pathways applying specific stimuli and recording the corresponding read-outs, i.e. expression of specific effectors or activation of STAT1 (Fig. 3; Additional file 10: Table S3). Consistent with the absence or reduced expression of TLR7 and TLR8 in permissive cell lines (Fig. 3a), stimulation of the TLR pathway with R848 failed to increase IL6 mRNA as measured by RT-qPCR, and in contrast to resting CD4+ T cells. As expected from the transcriptional integrity of the core STAT-dependent signaling of the IFN-gamma pathway, the addition of IFN-gamma to both resting CD4+ T cells and cell lines resulted in the successful phosphorylation of STAT1 (Additional file 11: Figure S8) and increased expression of IRF1 mRNA (Fig. 3b; Additional file 10: Table S3). IFN-gamma stimulation failed to result in detectable expression of IL1B mRNA in cell lines, consistent with low expression levels of key components in this cascade (e.g. IRF4; Additional file 8: Figure S6). In the case of the TNF-alpha signaling pathway, the integrity of the signaling cascade in cell lines at the transcriptional level was coherent with the detection of BIRC3 by RT-qPCR upon addition of TNF-alpha (Fig. 3b; Additional file 10: Table S3). However, only SupT1 cells displayed an increase of IL6.

### Expression of genes involved in HIV sensing and restriction

**Fig. 4:**
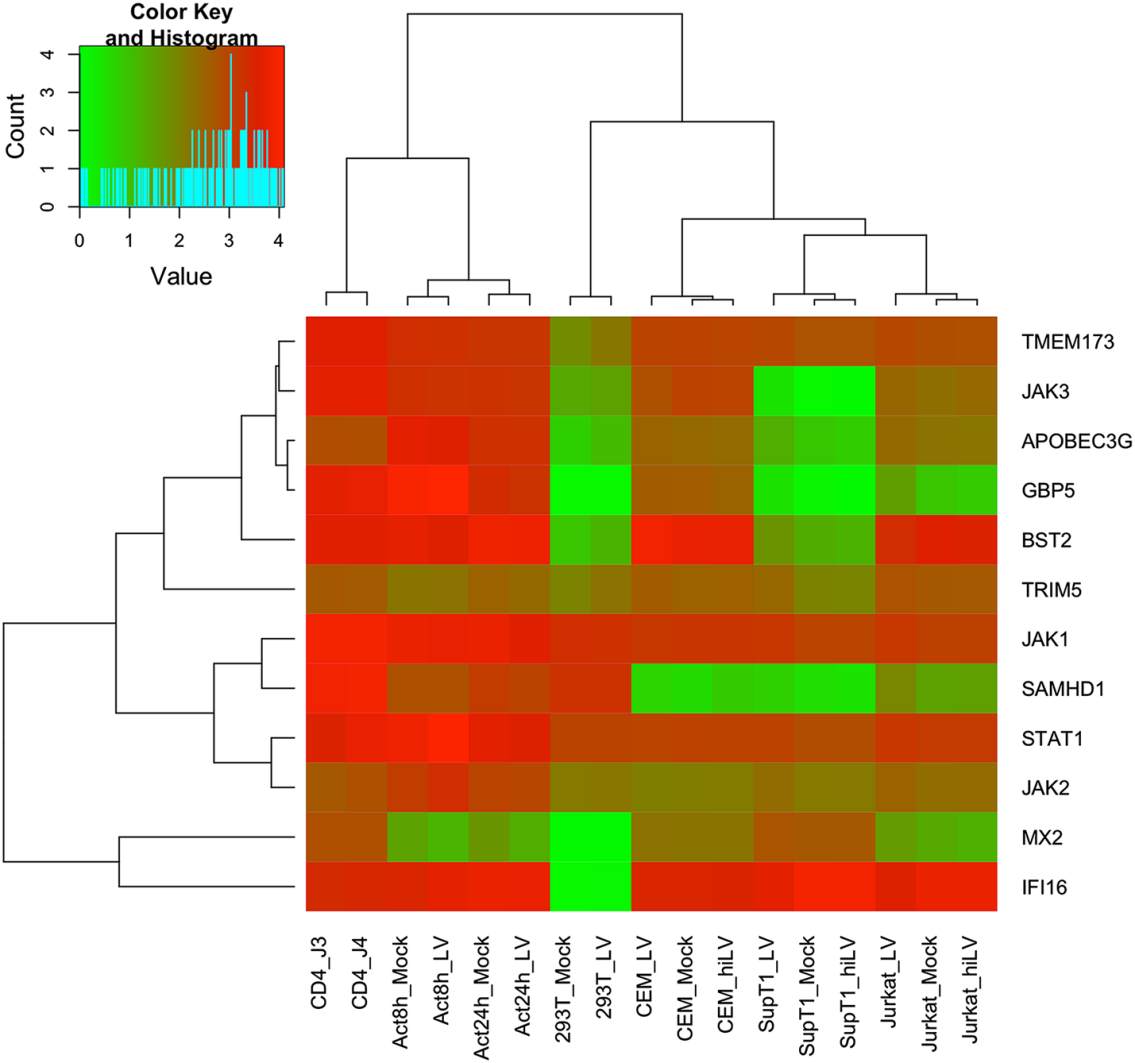
Heatmap of expression values of paradigmatic genes involved in antiretroviral defense and signaling relevant to HIV biology. The figure shows the expression values of antiretroviral genes (*APOBEC3G, TRIM5, BST2, MX2, GBP5,* and *SAMHD1*) and signaling genes (*JAK, STAT1, IFI16* and *STING/TMEM173*) in RNA-seq libraries of resting CD4+ T cells, cell lines HEK293T, Jurkat, SupT1 and CEM -mock (Mock), heat-inactivated (hiLV) and HIV-infected (LV)- and activated CD4+ T cells at 8 and 24 h after TCR activation (Methods). The *color-code scale* in the *inset* represents the expression levels (log10 transformation of the number of library size-normalized reads per kilobase of exonic sequence), ranging from *green* (low) to red (high) expression. Complete hierarchical clustering of genes was based on Pearson correlation of the expression levels. Complete hierarchical clustering of samples was kept as assessed in Additional file 6: Figure S4. JAK1, JAK2, TMEM173 and GBP5 had a fold change higher than 2 between resting CD4+ T cells and permissive cell lines (Benjamini–Hochberg adjusted p value <0.01, Methods)

Finally, we assessed the transcriptional pattern for paradigmatic genes involved in antiretroviral defense (*APOBEC3G, TRIM5, BST2, MX2, GBP5,* and *SAMHD1*) and signaling (*JAK, STAT1, IFI16* and *STING/TMEM173*) relevant to HIV biology (Fig. 4). Primary CD4+ T cells transcribed all those genes (expression levels ranging from 2.64 to 3.94 log10 reads per kilobase). In contrast, a diversity of patterns of reduced gene expression were observed across the cell lines: 293T (downregulation of *STING, JAK3, IFI16, APOBEC3G, GBP5, BST2, MX2*, and to a lesser extent other genes), SupT1 (downregulation of *JAK3, APOBEC3G, GBP5, BST2, SAMHD1*, and to a lesser extent other genes), Jurkat (downregulation of *GBP5, SAMHD1, MX2,* and to a lesser extent other genes), and CEM (downregulation of *SAMHD1* and to a lesser extent other genes). Globally, transcriptional data parallels protein expression levels and function across cell lines (www.proteinatlas.org and [19, 22–28]). Upon activation of primary CD4+ T cells, we observed a strong down-regulation of *MX2* and to a lesser extent *TRIM5*. These results suggest a pattern of focal defects in cell lines and in specific pathways that is consistent with the observations from the global analysis of innate immunity genes and pathways presented in the sections above.

## Conclusion

The innate immune response differs according to the cell type or cell state, such as activated vs resting CD4+ T cells, and this may in turn affect the outcome of viral infection [5, 29, 30]. Activated CD4+ T cells are more permissive to HIV infection in part because of reduced innate immune responses. This favors productive infection and virus-induced cell death by apoptosis. In contrast, resting CD4+ T cells are more resistant to HIV-1 infection, thanks to expression of innate immune defenses (SAMHD1-mediated impaired reverse transcription, IFI16-mediated viral nucleic acid sensing and signaling), leading to abortive infection and to cell death induced by pyroptosis (although this is not observed in vitro upon cell-free virus infection). Changes in expression of innate immunity signaling and effector molecules impact the model of cell death induced by HIV-1 infection, whether triggered by apoptosis or pyroptosis [29]. Therefore, the cell lines used to investigate viral infection may only partially reflect physiological innate immune responses.

Overall, our results show that permissive laboratory cell lines have transcriptional and functional defects in components of key innate immunity signaling pathways resulting in reduced activation or absence of effector gene expression upon specific stimulation. Such defects may contribute to the success of viral infection in cell lines compared to primary cells. This study supports the call for caution when investigating the interaction between viral and innate immunity factors using cell lines. Furthermore, it provides criteria for the choice of gain or loss of function screenings of viral infection; i.e., in the absence of expression of a target innate immunity factor, the best screen may be over-expression rather than knockdown.

## Methods

### Cell samples

#### Primary CD4+ T cells

Peripheral Blood Mononuclear Cells (PBMCs) from two different healthy blood donors were purified by Ficoll gradient separation followed by primary CD4+ T cell isolation using negative selection and magnetic separation (human CD4+ T Cell Isolation kit II; Miltenyi Biotec) as previously described [21] and directly used for total RNA extraction.

#### Activated CD4+ T cells

Activated CD4+ T cell data were from Mohammadi et al [21]. Briefly, primary CD4+ T cells isolated from a healthy blood donor were activated using CD3/CD28 co-stimulation in presence of IL-2 (mimicking T-Cell Receptor (TCR) stimulation). For this, anti-CD3 antibodies (10 μg) were plated in 1 ml PBS per well of a 6-well plate and incubated for 1–2 h at 37 °C. Wells were washed once with 3 ml of PBS and filled with 10^6^ cells/ml of primary CD4+ T cells supplemented with 1 μg/ml anti-CD28 antibodies in R-10 culture medium containing 100 U/ml human recombinant IL-2 (R&D Systems). Three days post-stimulation, activated CD4+ T cells (10^6^ cells) were infected or not with 5 μg p24 equivalent of HIVeGFP/VSV-G particles in presence of 2.5 μg/ml polybrene and in 300 μl final volume by spinoculation (1500 g, 25 °c, 2 h). Cells were left to return to a resting state by co-culture on a feeder cell layer for 10 weeks. Mock or infected resting CD4+ T cells were re-activated by TCR stimulation for 8 and 24 h before total RNA extraction.

#### Cell lines

Three T lymphoblastic cell lines, SupT1, Jurkat and CEM cells were cultured in RPMI 1640 (Invitrogen) supplemented with 10 % FCS and 50 μg/ml gentamicin (R-10 culture medium). Human embryonic kidney (HEK) 293T cells were cultured in Dulbecco’s Modified Eagle Medium (DMEM; Invitrogen) supplemented with 10 % heat-inactivated fetal calf serum (FCS) and 50 μg/ml gentamicin (D-10 culture medium).

Cell lines were infected or not by HIVeGFP/VSV-G for 24 h before total RNA extraction. Briefly HEK293T cells (200,000 cells) were plated in 12-well plates in 2 ml culture medium and let adhere over night before infection by spinoculation (1500 g, 25 °C, 3 h) with 1 μg p24 equivalent of HIVeGFP/VSV-G particles in presence of 20 μg/ml polybrene. SupT1, Jurkat and CEM T cell lines (10^6^ cells) were infected with 3 μg p24 equivalent of HIVeGFP/VSV-G particles in presence of 5 μg/ml polybrene and in 400 μl final volume by spinoculation (1500 g, 25 °c, 2 h). As controls, mock infection as well as infection using HIVeGFP/VSV-G heat-inactivated at 56 °C for 60 min were performed in parallel.

#### HIV-based vector production

HEK293T cells were co-transfected with 15 μg pNL4-3ΔEnv-GFP (NIH AIDS Research and Reference Reagent program, Cat. #11100; [31]) and 5 μg pMD.G [32], using the calcium phosphate method (Invitrogen) to generate HIVeGFP/VSV-G particles as described previously [3]. HIV titer was measured by p24 ELISA (Abbott).

### RNA seq and bioinformatic analyses

Cell lines infected with HIV-based vector, or mock infected, were collected for RNA extraction (Illustra RNAspin mini isolation kit; GE Healthcare) and subsequent mRNA-Seq transcriptome analysis as described previously [21]. mRNA-Seq library preparation was done with TruSeq RNA sample prep kit (Illumina) starting with capture of polyA-containing transcripts, followed by cluster generation (TruSeq cluster generation kit, Illumina) and high-throughput sequencing on Illumina HiSeq2000 at the Genomics Technology Facility, University of Lausanne. Single read 100 base pairs were performed in all libraries except for primary CD4+ T cells (samples CD4J3 and CD4J4) that were paired-end 100 base pairs. Sequencing data were bioinformatically analyzed as if they were single-end. The 100 bp single-end reads were trimmed and filtered before alignment as described in [21]. RNA-Seq data for activated cells (TCR-stimulated for 8 and 24 h) were from Mohammadi et al. (PLoS Pathogens 2014). Filtered reads were aligned to the human reference genome with RUM aligner (version v2.0.4; [33]) using the Ensembl gene GRCh37 release 70 annotation file concatenated to the HIV vector sequence used for infection. The number of reads per gene was quantified with HTSeq-count v.0.6.1 [34] with parameters mode = union and type = exon. We obtained an average library size of 77,999,650 uniquely mapped reads. When indicated in downstream analysis, log-transformation of gene expression values was performed as the log10 of the number of library size-normalized reads per kilobase of exonic sequence. A pseudo-count of 1 was added prior to the log10 transformation to avoid NA’s: log10(RPKM*77999650/1,000,000+1); RPKM: Reads per Kilobase per Million mapped Reads. Variance stabilization transformation was performed with R package DESeq [35] with parameters method “blind” for the computation of the empirical dispersion and fitType = “local” for fitting a dispersion-mean relation. The per-gene raw read-counts matrix and the RPKM matrix are provided as Additional files 12 and 13: Tables S4 and S5, respectively (see also Additional file 14: Table S6). Differential expression analysis between primary cells and cell lines used DESeq2 package [36]. An R script with all statistical analyses performed necessary to reproduce results and figures is provided as Additional file 15: File S1. Versions of R-packages used are detailed in Additional file 16: File S2.

### Perimeter of innate immunity genes

A representative list of 1503 human innate immunity genes compiled as described in [20] was used (Additional file 1: Table S1). The list represents the union of four resources: (1) genes annotated with the term “innate immunity response” (GO:0045087) in the Gene Ontology project (http://www.geneontology.org/) [37]; (2) innate immunity genes manually annotated in the InnateDB database (http://www.innatedb.ca) [38]; (3) IFN-stimulated genes from the ISG database [39] identified through expression analyses, and (4) a list of IFN-stimulated genes used for extensive functional analyses in the context of viral infection, including HIV [40].1473 of the 1503 innate immunity genes were found expressed in at least one of the resting CD4+ T cell samples or laboratory cell lines analyzed.

### Functional analyses

#### Functional analysis of innate immunity pathway

SupT1, Jurkat and CEM T cells lines (10^6^ cells) were seeded in 12-well plates in 1 ml R-10 culture medium in presence of two concentrations of compound. Functional analyses used 1 and 10 μg/ml R848 (ligand of TLR7/8), 0.1 and 1 μM CpG oligodeoxynucleotide (ligand of TLR9), 10 and 100 U/ml IFN-γ, 5 and 25 ng/ml TNF, and 1 and 10 μg/ml MDP (ligand of NOD2). Mock treatment was used as negative control. After 1, 4 and 24 h of incubation, cells were collected for downstream analyses: total RNA extraction (Illustra RNAspin mini isolation kit; GE Healthcare) and gene expression analysis by RT-qPCR, or for cell lysis and immunoblot analysis. Primary resting CD4+ T cells (10^6^ cells) isolated from two healthy blood donors were tested similarly in R-10 culture medium only (negative control), in presence of 10 μg/ml R848, 100 and 1000 U/ml IFN-γ, or TCR stimulation (anti-CD3/anti-CD28/IL2) for 24 h (Additional files 17 and 18: File S3 and S4).

#### Gene expression analysis

RNA (200 ng) was reverse transcribed using High-Capacity cDNA Reverse Transcription (Life Technologies) according to manufacturer’s instructions in a total volume of 20 μl. Eight genes representative of the selective pathways were assessed in duplicate by qPCR using 2 μl cDNA, and commercially available Gene Expression Assays with FAM-MGB probes (Applied Biosystems) following manufacturer’s recommendations: IL1B (Hs01555410_m1), IRF1 (Hs00971960_m1), IFNB1 (Hs01077958_s1), IL12B (Hs01011518_m1), IL6 (Hs00985639_m1), CASP1 (Hs00236158_m1), IL18 (Hs01038788_m1) and BIRC3 (Hs00985031_g1). PIGS mRNA with a VIC-MGB probe (Hs00264209_m1) was used as endogenous control. qPCR was carried out in a StepOnePlus (Applied Biosystems) using the following cycling conditions: 2′ at 50 °C, 10′ at 95 °C, 40 cycles of 15″ at 95 °C and 1’ at 60 °C. Calculations were ΔΔC_T_ = (C_T_ gene − C_T_ PIGS)_compound_ − (C_T_ gene – C_T_ PIGS)_mock_. Log2 fold change of RT-qPCR data of compound over mock treated samples corresponds to the −ΔΔCT.

#### Immunoblot analysis

Cells (10^6^) were lysed in RIPA buffer (50 mM Tris-HCl [pH 8.0], 150 mM NaCl, 2 mM EDTA, 1 % NP-40, 0.1 % SDS, 0.5 % sodium deoxycholate) supplemented with protease inhibitors (Complete Mini; Roche) and phosphatase inhibitors (PhosStop; Roche) for 45′ at 4 °C. Cell lysates were centrifuged and clean supernatants were further used for protein quantification (BCA Protein Assay kit; Pierce) following manufacturer’s instructions. Whole cell lysates (10 μg total proteins) were separated by SDS-polyacrylamide gel electrophoresis and transferred to a nitrocellulose membrane. Total phospho-Stat1 (Mouse anti-P-Y701 Stat1, 1:1000, #612132, BD Biosciences) and Stat1 (Rabbit anti-Stat1 antibody, 1:1000, #9172, Primary Cell Signaling Technology) were detected using standard procedures with Tris-Buffered Saline (TBS)-0.2 % Tween-5 % BSA and PBS-0.2 % Tween-5 % milk as blocking buffers for Phospho-Stat1 and Stat1 respectively. Secondary antibodies were rabbit anti-mouse-HRP (1:5000, #P0260, DAKO) and swine anti-rabbit-HRP (1:1000, #P02117, DAKO) respectively. Membrane stripping was performed according to Blot Restore Membrane Rejuvination kit (Millipore) followed by tubulin detection (Mouse anti-tubulin, 1:10,000, #T5168, Sigma). Detection was finalized using ECL chemiluminescence detection (LiteAblot; Euroclone).

## Additional files

10.1186/s12977-016-0275-8 List of 1503 innate immunity genes used in this study.
10.1186/s12977-016-0275-8 Principal component analysis of RNA-Seq libraries. The figure shows the first two principal axes of the whole transcriptome Principal Component Analysis (PCA) analysis of resting CD4+ T cells (dark red) from two donors and four human laboratory cell lines HEK293T (pink), Jurkat (light violet), SupT1 (magenta) and CEM (dark violet). Cell lines were evaluated in 3 conditions: uninfected mock (circles), infected with a heat-inactivated HIV vector (squares) and HIV-infected (triangles). The percentage of variance explained by each axis is indicated. PCA was performed on the variance-stabilized transformation of read counts as described in Methods. The first principal axis (PCA1) of the PCA of the RNA-Seq libraries separated samples according to their permissiveness to HIV infection, from primary resting CD4+ T cells to permissive cell lines. The second principal axis distinguished lymphoblastic versus non-lymphoblastic cell lineages (PC2). Although HIV infection is known to modify the cellular transcriptome [3] it does not appear as a main factor of the distribution on the PCA space. Indeed, libraries of the same cell line, either in infected or uninfected conditions, clustered together, showing that main transcriptional differences are driven by cell type and not by infection state consistent with previous studies [21].
10.1186/s12977-016-0275-8 Description of the genes in the 249 and 110 clusters. A. List of 249 innate immunity genes that were had high expression in primary cells and low or absent expression in cell lines. B. List of 110 innate immunity genes that with low expression in resting CD4+ T cells and high expression in cell lines. The DESeq2 results of the differential expression analysis between primary CD4+ T cells and cell lines are indicated.
10.1186/s12977-016-0275-8 Heatmap of expression values of innate immunity genes in resting CD4+ T cells, and laboratory cell lines in a uninfected state. The figure shows the expression values of 1473 innate immunity genes in the samples shown in Figure 1 excluding samples heat-inactivated (HI) and HIV-infected (HIV). Complete hierarchical clustering of genes and cell samples was based on Pearson correlation of variance-stabilized read counts (Methods). Color scale indicated in the legend corresponds to z-scores of RPKM distributions per gene, ranging from green (low) to red (high) expression. The genes belonging to the 249 genes and 110 genes clusters detected in Figure 1 are indicated in the left side of the heatmap as stripes colored in dark blue and cyan respectively.
10.1186/s12977-016-0275-8 Principal component analysis of RNA-Seq libraries including activated CD4+ T cells. The figure shows the first two principal axes of the whole transcriptome Principal Component Analysis (PCA) analysis of the 13 samples shown in Supplemental Figure 1 plus 4 samples corresponding to Activated CD4+ T cells at 8h (red) and 24h (orange) after TCR activation. The percentage of variance explained by each axis is indicated. PCA was performed on the variance-stabilized transformation of read counts as described in Methods. As in Supplemental Figure 1, the first principal axis (PC1) of the PCA separated samples according to their permissiveness to HIV infection. Thus, activated CD4+ T cells mapped in an intermediate position along PC1, between primary resting CD4+ T cells and permissive cell lines.
10.1186/s12977-016-0275-8 Heatmap of expression values of innate immunity genes in resting CD4+ T cells, activated CD4+ T cells and laboratory cell lines. The figure shows the expression values of 1473 innate immunity genes in the 13 samples shown in Figure 1 plus 4 samples corresponding to Activated CD4+ T cells at 8 and 24h after TCR activation. Complete hierarchical clustering of genes and cell samples was based on Pearson correlation of variance-stabilized read counts (Methods). Color scale indicated in the legend corresponds to z-scores of RPKM distributions per gene, ranging from green (low) to red (high) expression. The genes belonging to the 249 genes and 110 genes clusters detected in Figure 1 are indicated in the left side of the heatmap as stripes colored in dark blue and cyan respectively.
10.1186/s12977-016-0275-8 Toll-like receptor (TLR) pathway. Representation of the TLR pathway taken from WikiPathways database [41]. Boxes representing genes display the transcriptional levels detected in RNA-seq libraries of resting CD4+ T cells, and the four human laboratory cell lines HEK293T, Jurkat, SupT1 and CEM -mock (MO), heat-inactivated (HI) and HIV-infected (HIV)- and 4 samples corresponding to Activated CD4+ T cells at 8h and 24h after TCR activation, following the same order of the libraries and color-code scale of expression levels as indicated in Figure 3A. The figure was generated using Pathvisio-3 software [42].
10.1186/s12977-016-0275-8 Interferon (IFN) gamma signaling pathway. Representation of the IFN gamma signaling pathway taken from WikiPathways database [41]. Boxes representing genes display the transcriptional levels detected in RNA-seq libraries of resting CD4+ T cells, and the four human laboratory cell lines HEK293T, Jurkat, SupT1 and CEM -mock (MO), heat-inactivated (HI) and HIV-infected (HIV)- and 4 samples corresponding to Activated CD4+ T cells at 8h and 24h after TCR activation, following the same order of the libraries and color-code scale of expression levels as indicated in Figure 3A. The figure was generated using Pathvisio-3 software [42].
10.1186/s12977-016-0275-8 TNF-alpha signaling pathway. Representation of the TNF-alpha signaling pathway taken from WikiPathways database [41]. Boxes representing genes display the transcriptional levels detected in RNA-seq libraries of resting CD4+ T cells, and the four human laboratory cell lines HEK293T, Jurkat, SupT1 and CEM -mock (MO), heat-inactivated (HI) and HIV-infected (HIV)- and 4 samples corresponding to Activated CD4+ T cells at 8h and 24h after TCR activation, following the same order of the libraries and color-code scale of expression levels as indicated in Figure 3A. The figure was generated using Pathvisio-3 software [42].
10.1186/s12977-016-0275-8 RT-qPCR results.
10.1186/s12977-016-0275-8 IFN-γ-induced phosphorylation of Stat1. (A) T cell lines (CEM, Jurkat, SupT1) were mock-stimulated (C-) or stimulated using 100 U IFN-γ for different times (1h, 4h, 24h), before cell lysis. Immunoblot analysis of whole cell lysates was performed using anti-phosphorylated Stat1, anti-Stat1 or anti-α-Tubulin detection. (B) Resting CD4+ T cells isolated from two healthy blood donors were either mock- treated (C-), simulated with 100 U IFN-γ or, stimulated using anti-CD3/anti-CD28/IL2 (TCR) as positive control (C+) for 24h before immunoblot analysis as in A.
10.1186/s12977-016-0275-8 Raw read counts per library of the RNA-seq libraries sequenced in this work.
10.1186/s12977-016-0275-8 RPKMs of the RNA-seq libraries sequenced in this work.
10.1186/s12977-016-0275-8 Table describing RNA-seq libraries that is used as an input for DESeq2 functions in Supplemental File 1.
10.1186/s12977-016-0275-8 R script gathering the different statistical analyses needed to reproduce the main figures and results of the paper.
10.1186/s12977-016-0275-8 R package versions used during the execution of Supplemental File 2.
10.1186/s12977-016-0275-8 List of the 249-cluster used as input in Supplemental File 1.
10.1186/s12977-016-0275-8 List of the 110-cluster used as input in Supplemental File 1.
