# Supplementary figures and images for: Innate immune defects in HIV permissive cell lines
Source: Retrovirology. 2016 Jun 27;13:43. doi: 10.1186/s12977-016-0275-8 (PMC4924258; doi:10.1186/s12977-016-0275-8)

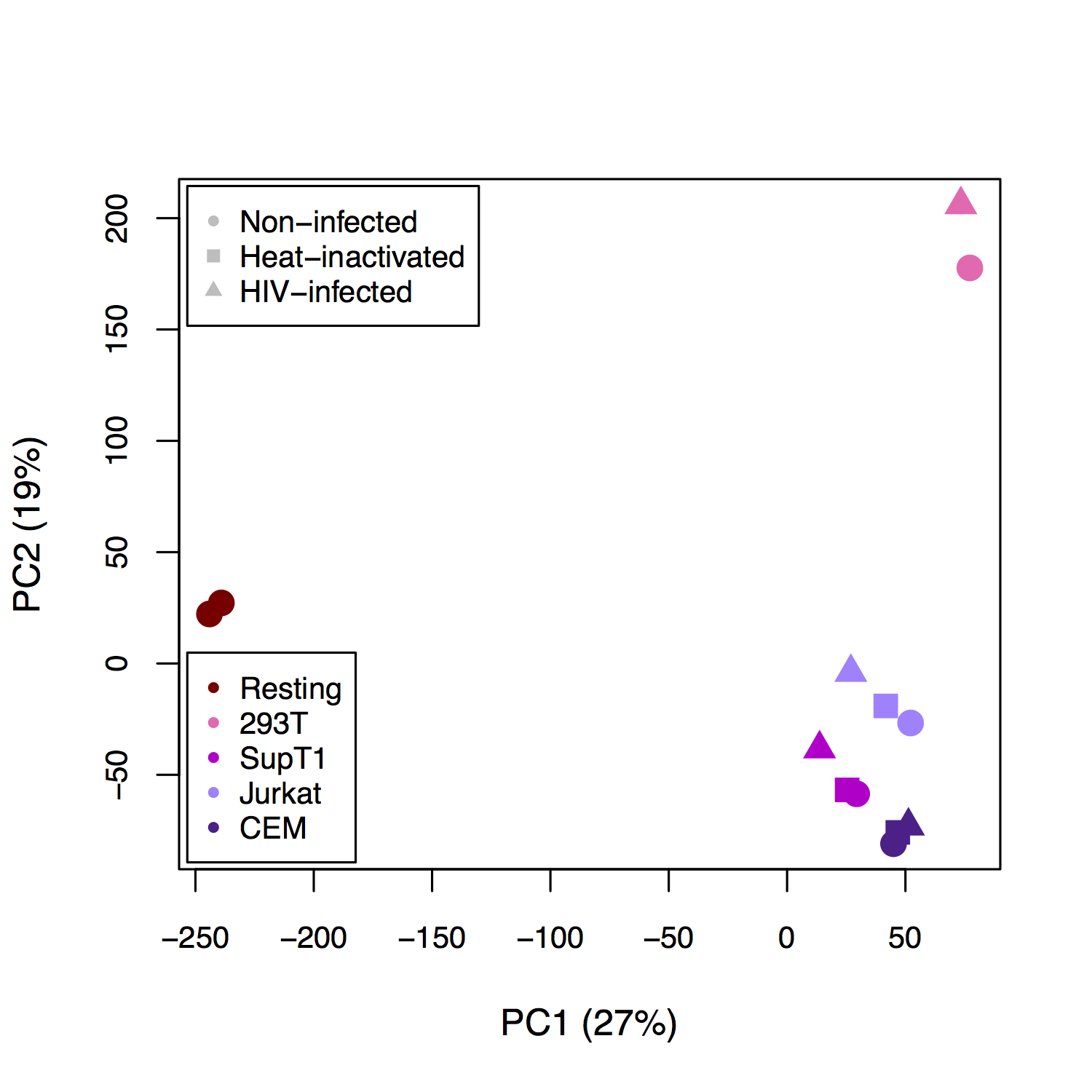

Supplement: Supplementary file 2 — 10.1186/s12977-016-0275-8 Principal component analysis of RNA-Seq libraries. The figure shows the first two principal axes of the whole transcriptome Principal Component Analysis (PCA) analysis of resting CD4+ T cells (dark red) from two donors and four human laboratory cell lines HEK293T (pink), Jurkat (light violet), SupT1 (magenta) and CEM (dark violet). Cell lines were evaluated in 3 conditions: uninfected mock (circles), infected with a heat-inactivated HIV vector (squares) and HIV-infected (triangles). The percentage of variance explained by each axis is indicated. PCA was performed on the variance-stabilized transformation of read counts as described in Methods. The first principal axis (PCA1) of the PCA of the RNA-Seq libraries separated samples according to their permissiveness to HIV infection, from primary resting CD4+ T cells to permissive cell lines. The second principal axis distinguished lymphoblastic versus non-lymphoblastic cell lineages (PC2). Although HIV infection is known to modify the cellular transcriptome [3] it does not appear as a main factor of the distribution on the PCA space. Indeed, libraries of the same cell line, either in infected or uninfected conditions, clustered together, showing that main transcriptional differences are driven by cell type and not by infection state consistent with previous studies [21]. [file 12977_2016_275_MOESM2_ESM.tiff]

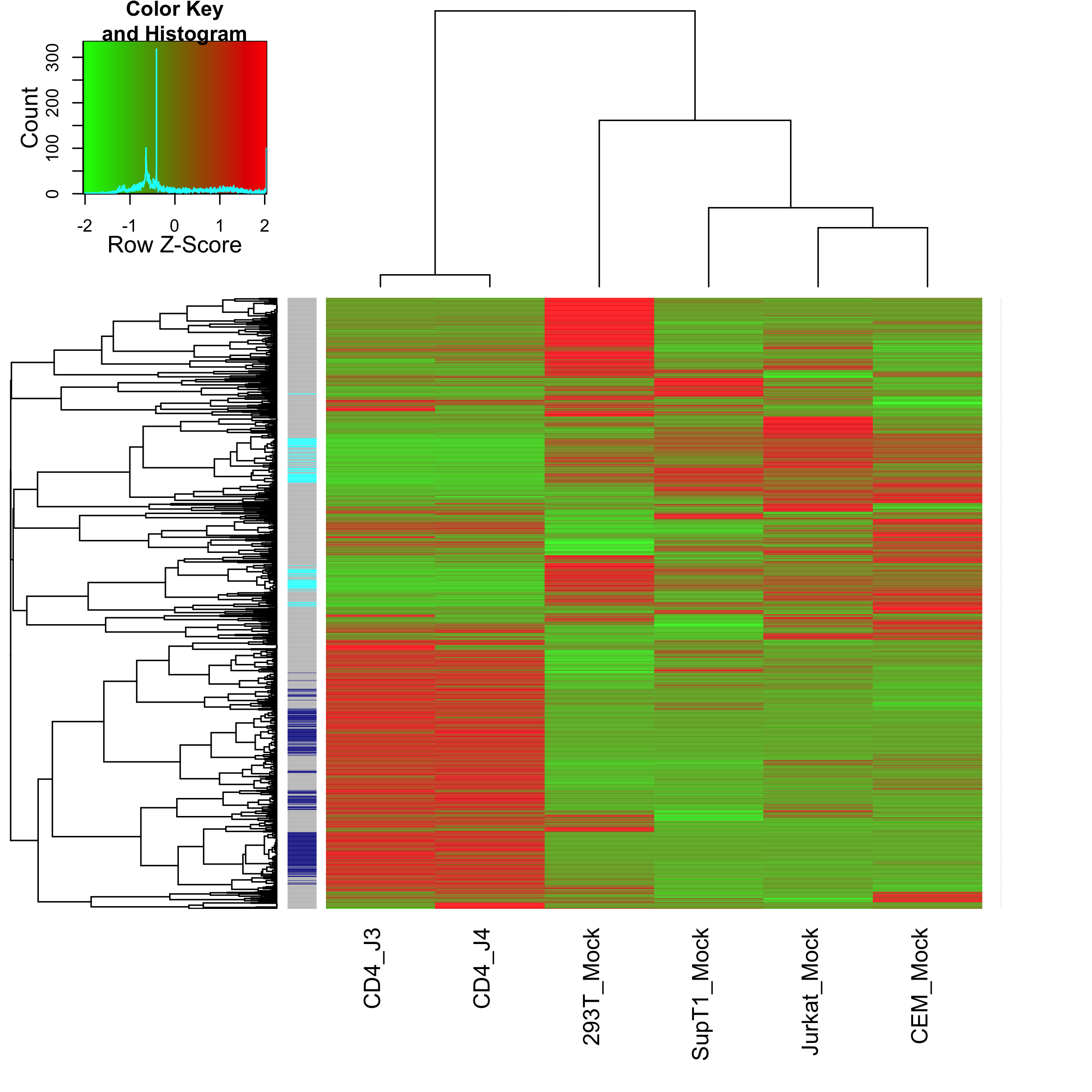

Supplement: Supplementary file 4 — 10.1186/s12977-016-0275-8 Heatmap of expression values of innate immunity genes in resting CD4+ T cells, and laboratory cell lines in a uninfected state. The figure shows the expression values of 1473 innate immunity genes in the samples shown in Figure 1 excluding samples heat-inactivated (HI) and HIV-infected (HIV). Complete hierarchical clustering of genes and cell samples was based on Pearson correlation of variance-stabilized read counts (Methods). Color scale indicated in the legend corresponds to z-scores of RPKM distributions per gene, ranging from green (low) to red (high) expression. The genes belonging to the 249 genes and 110 genes clusters detected in Figure 1 are indicated in the left side of the heatmap as stripes colored in dark blue and cyan respectively. [file 12977_2016_275_MOESM4_ESM.tiff]

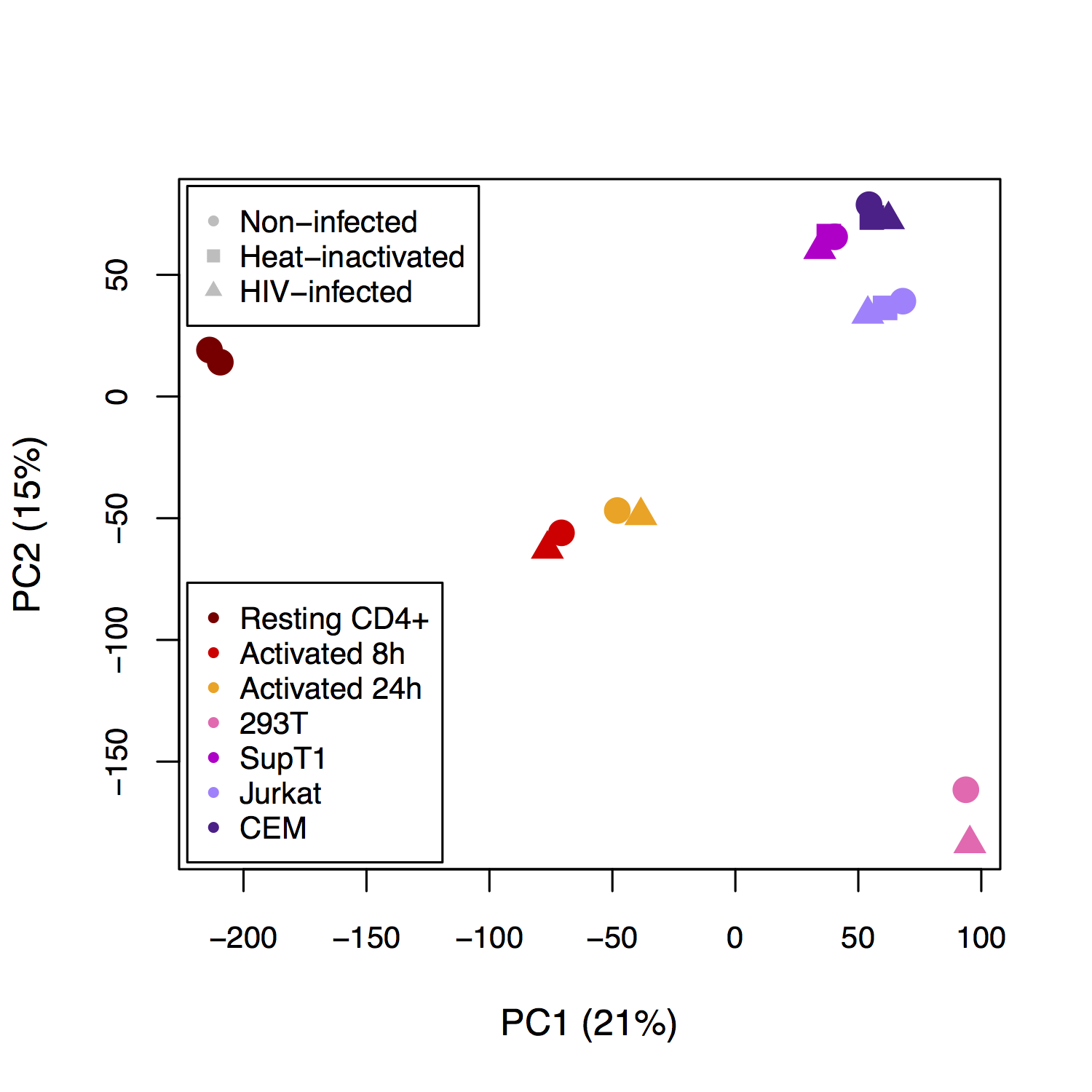

Supplement: Supplementary file 5 — 10.1186/s12977-016-0275-8 Principal component analysis of RNA-Seq libraries including activated CD4+ T cells. The figure shows the first two principal axes of the whole transcriptome Principal Component Analysis (PCA) analysis of the 13 samples shown in Supplemental Figure 1 plus 4 samples corresponding to Activated CD4+ T cells at 8h (red) and 24h (orange) after TCR activation. The percentage of variance explained by each axis is indicated. PCA was performed on the variance-stabilized transformation of read counts as described in Methods. As in Supplemental Figure 1, the first principal axis (PC1) of the PCA separated samples according to their permissiveness to HIV infection. Thus, activated CD4+ T cells mapped in an intermediate position along PC1, between primary resting CD4+ T cells and permissive cell lines. [file 12977_2016_275_MOESM5_ESM.tiff]

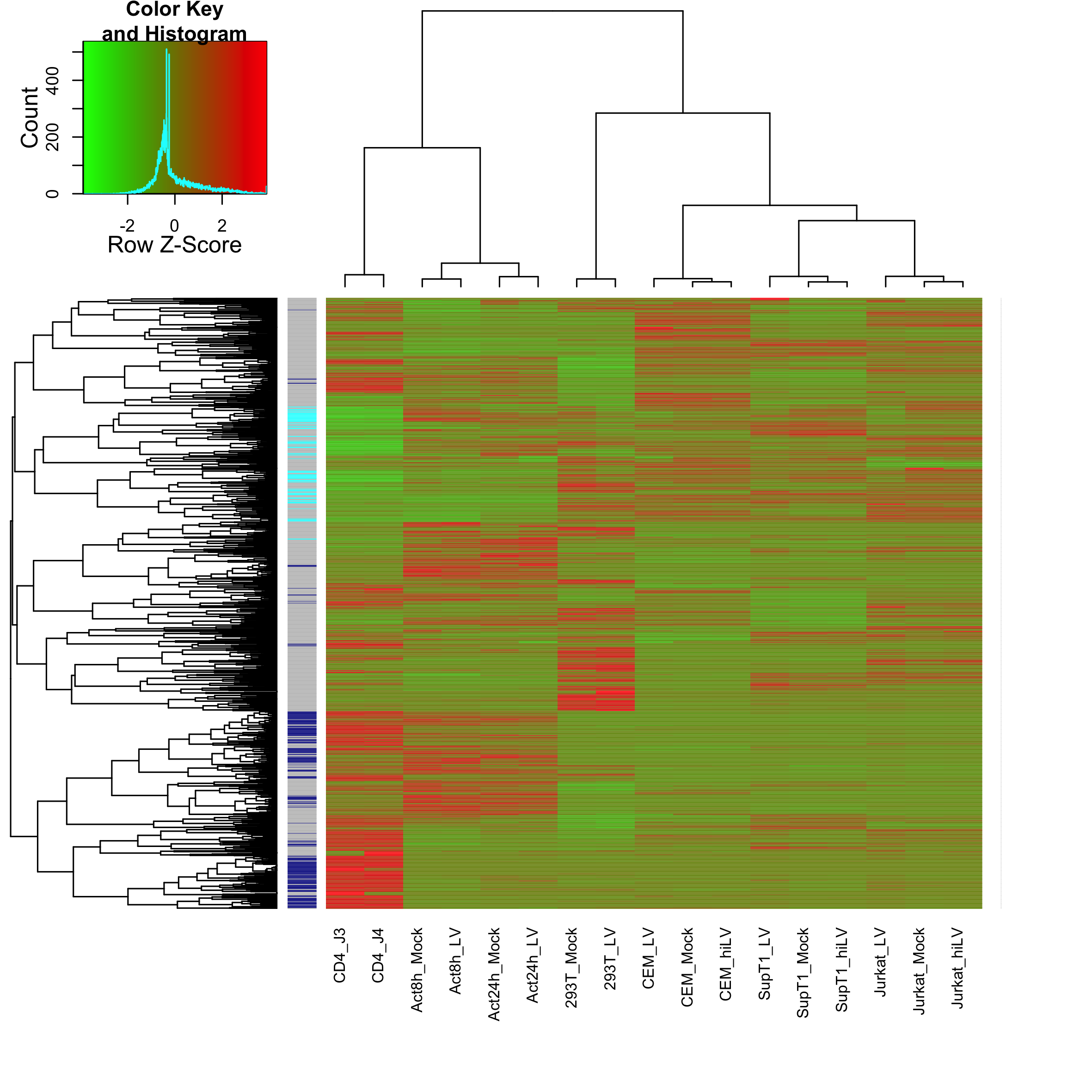

Supplement: Supplementary file 6 — 10.1186/s12977-016-0275-8 Heatmap of expression values of innate immunity genes in resting CD4+ T cells, activated CD4+ T cells and laboratory cell lines. The figure shows the expression values of 1473 innate immunity genes in the 13 samples shown in Figure 1 plus 4 samples corresponding to Activated CD4+ T cells at 8 and 24h after TCR activation. Complete hierarchical clustering of genes and cell samples was based on Pearson correlation of variance-stabilized read counts (Methods). Color scale indicated in the legend corresponds to z-scores of RPKM distributions per gene, ranging from green (low) to red (high) expression. The genes belonging to the 249 genes and 110 genes clusters detected in Figure 1 are indicated in the left side of the heatmap as stripes colored in dark blue and cyan respectively. [file 12977_2016_275_MOESM6_ESM.tiff]

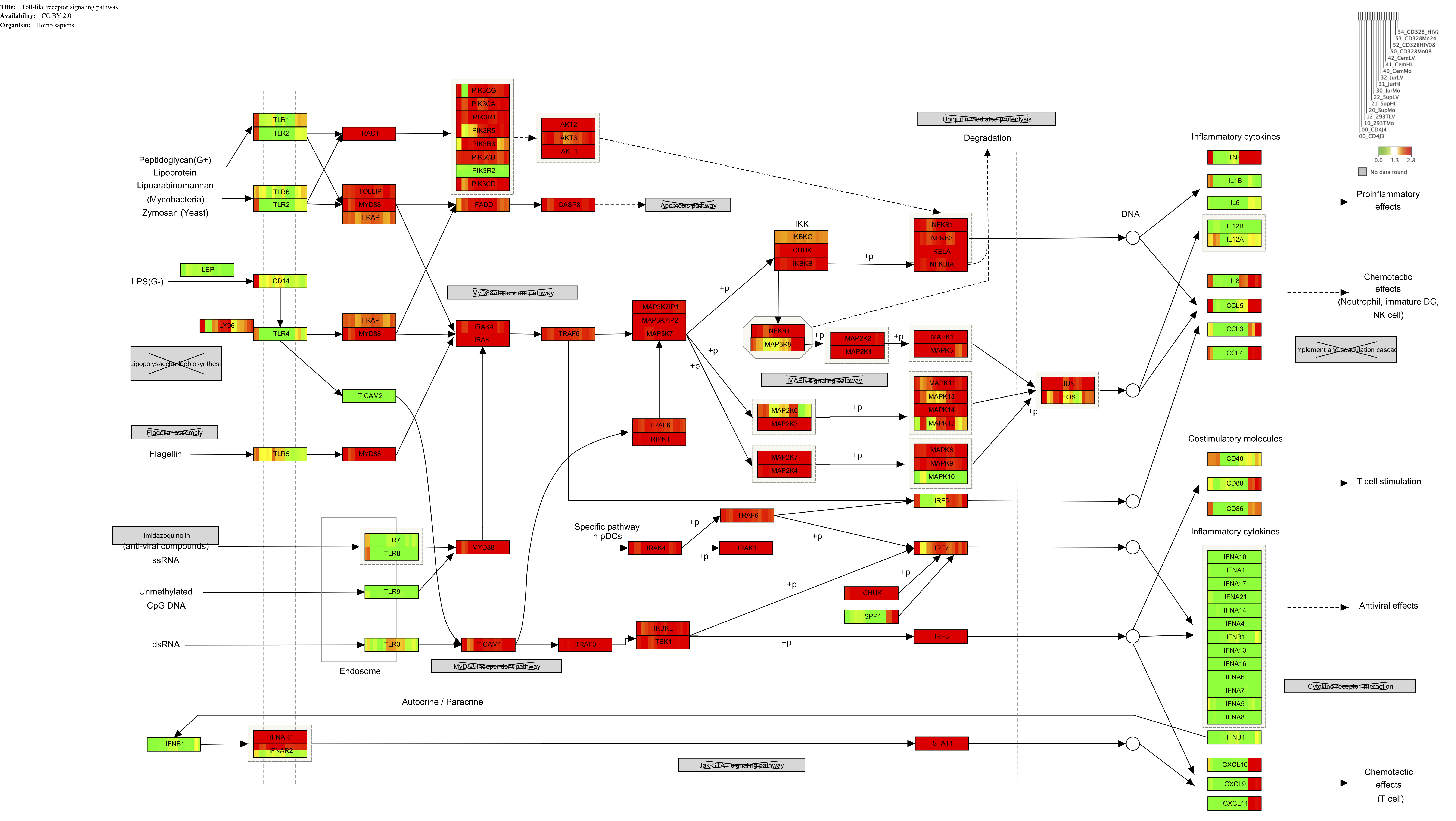

Supplement: Supplementary file 7 — 10.1186/s12977-016-0275-8 Toll-like receptor (TLR) pathway. Representation of the TLR pathway taken from WikiPathways database [41]. Boxes representing genes display the transcriptional levels detected in RNA-seq libraries of resting CD4+ T cells, and the four human laboratory cell lines HEK293T, Jurkat, SupT1 and CEM -mock (MO), heat-inactivated (HI) and HIV-infected (HIV)- and 4 samples corresponding to Activated CD4+ T cells at 8h and 24h after TCR activation, following the same order of the libraries and color-code scale of expression levels as indicated in Figure 3A. The figure was generated using Pathvisio-3 software [42]. [file 12977_2016_275_MOESM7_ESM.tiff]

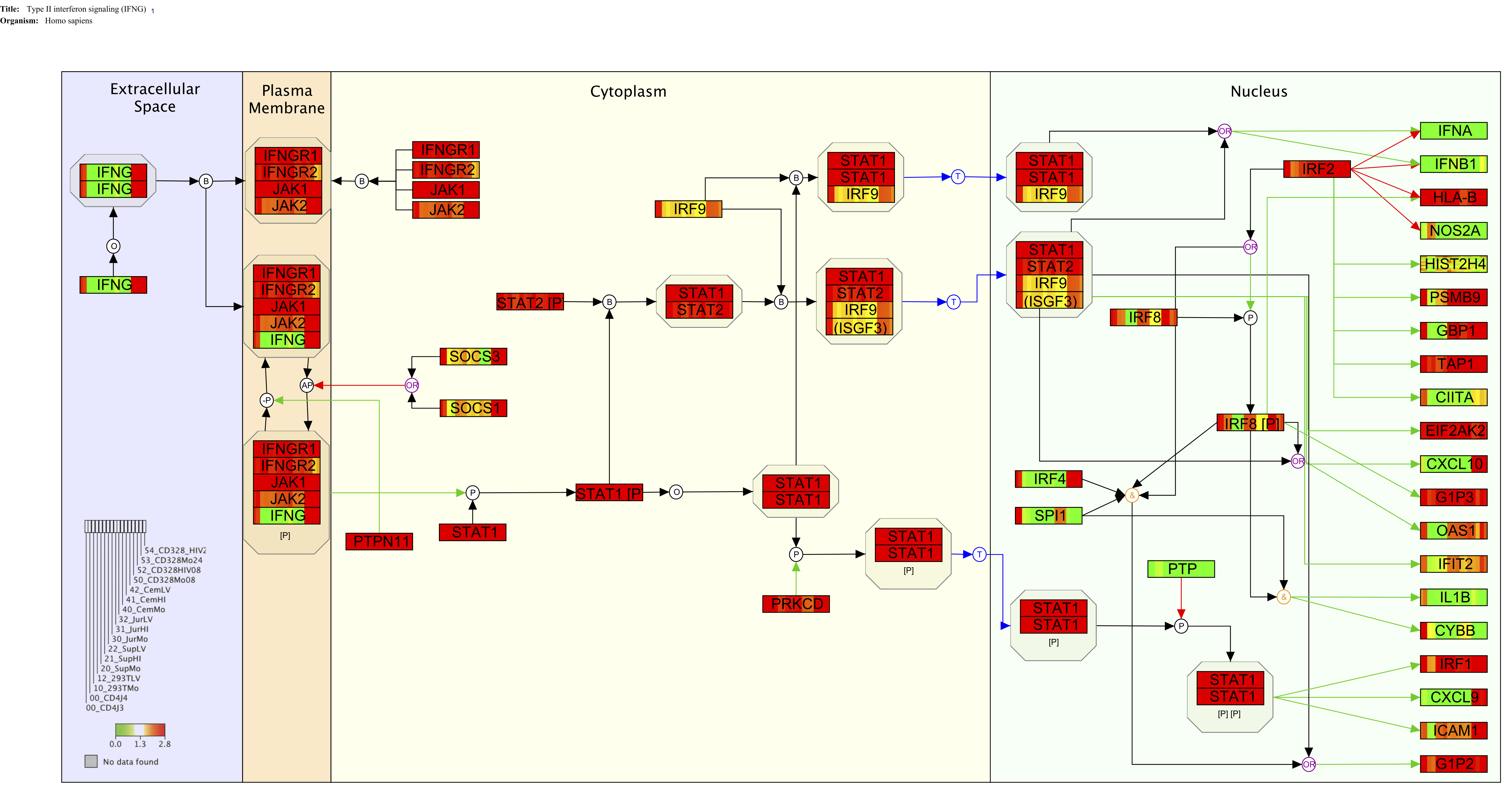

Supplement: Supplementary file 8 — 10.1186/s12977-016-0275-8 Interferon (IFN) gamma signaling pathway. Representation of the IFN gamma signaling pathway taken from WikiPathways database [41]. Boxes representing genes display the transcriptional levels detected in RNA-seq libraries of resting CD4+ T cells, and the four human laboratory cell lines HEK293T, Jurkat, SupT1 and CEM -mock (MO), heat-inactivated (HI) and HIV-infected (HIV)- and 4 samples corresponding to Activated CD4+ T cells at 8h and 24h after TCR activation, following the same order of the libraries and color-code scale of expression levels as indicated in Figure 3A. The figure was generated using Pathvisio-3 software [42]. [file 12977_2016_275_MOESM8_ESM.tiff]

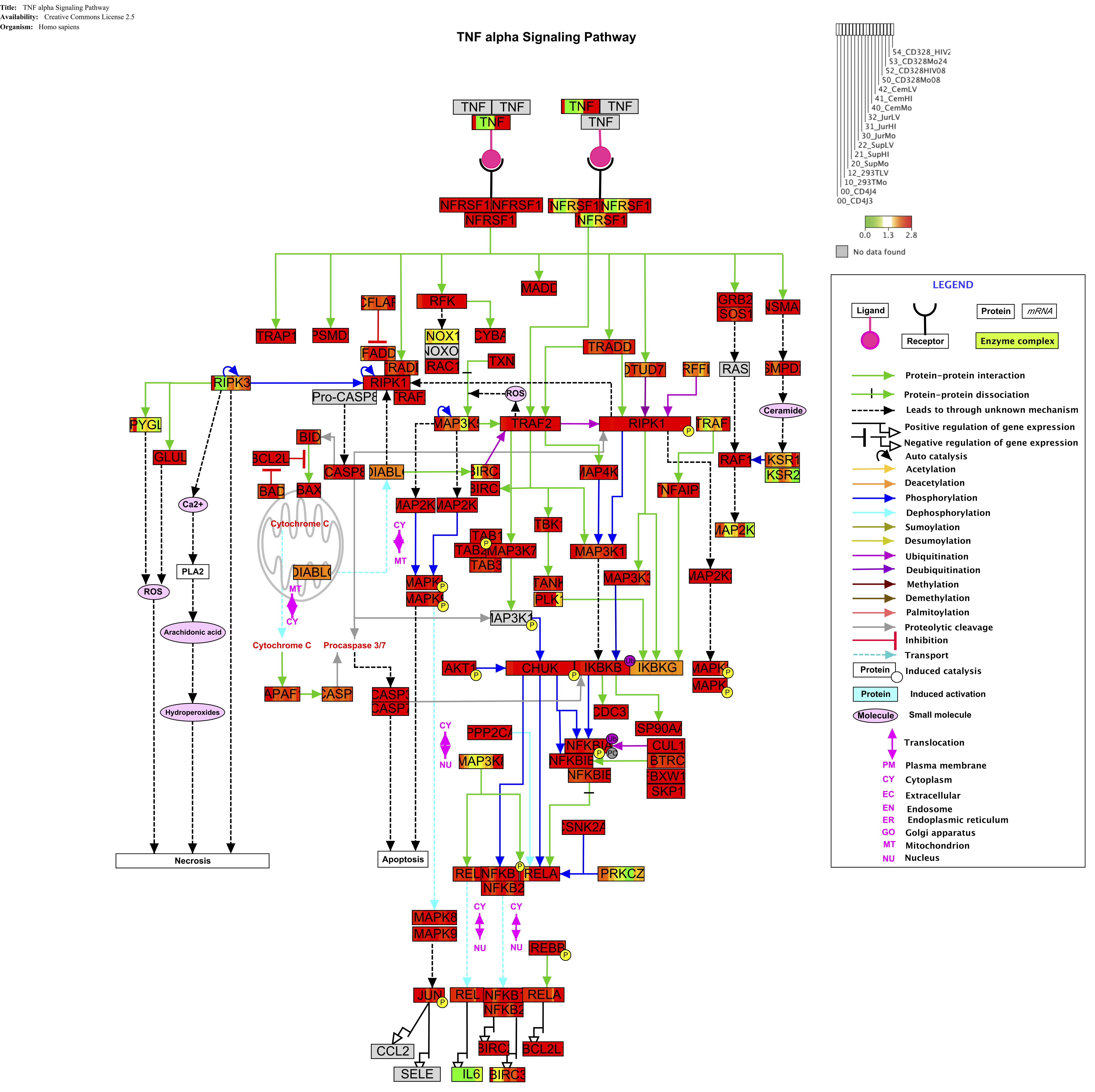

Supplement: Supplementary file 9 — 10.1186/s12977-016-0275-8 TNF-alpha signaling pathway. Representation of the TNF-alpha signaling pathway taken from WikiPathways database [41]. Boxes representing genes display the transcriptional levels detected in RNA-seq libraries of resting CD4+ T cells, and the four human laboratory cell lines HEK293T, Jurkat, SupT1 and CEM -mock (MO), heat-inactivated (HI) and HIV-infected (HIV)- and 4 samples corresponding to Activated CD4+ T cells at 8h and 24h after TCR activation, following the same order of the libraries and color-code scale of expression levels as indicated in Figure 3A. The figure was generated using Pathvisio-3 software [42]. [file 12977_2016_275_MOESM9_ESM.tiff]

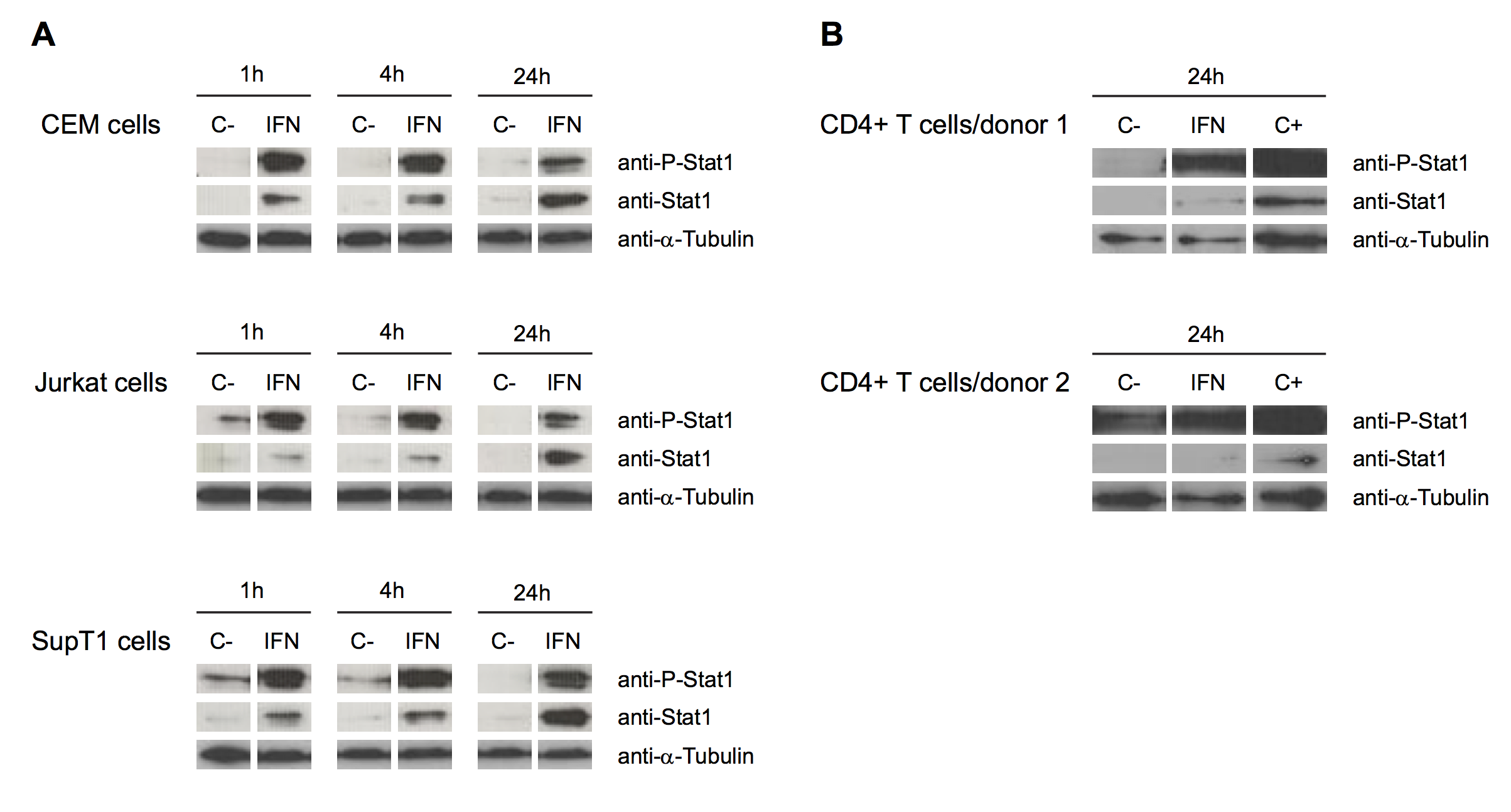

Supplement: Supplementary file 11 — 10.1186/s12977-016-0275-8 IFN-γ-induced phosphorylation of Stat1. (A) T cell lines (CEM, Jurkat, SupT1) were mock-stimulated (C-) or stimulated using 100 U IFN-γ for different times (1h, 4h, 24h), before cell lysis. Immunoblot analysis of whole cell lysates was performed using anti-phosphorylated Stat1, anti-Stat1 or anti-α-Tubulin detection. (B) Resting CD4+ T cells isolated from two healthy blood donors were either mock- treated (C-), simulated with 100 U IFN-γ or, stimulated using anti-CD3/anti-CD28/IL2 (TCR) as positive control (C+) for 24h before immunoblot analysis as in A. [file 12977_2016_275_MOESM11_ESM.tiff]
